# National scale of neonatal CPAP to district hospitals in Malawi improves survival for neonates weighing between 1.0 and 1.3 kg

**DOI:** 10.1136/archdischild-2021-322964

**Published:** 2021-11-01

**Authors:** Jennifer Carns, Sara Liaghati-Mobarhan, Aba Asibon, Alfred Chalira, Norman Lufesi, Elizabeth Molyneux, Maria Z Oden, Rebecca Richards-Kortum, Kondwani Kawaza

**Affiliations:** 1 Rice360 Institute for Global Health, Rice University, Houston, Texas, USA; 2 Department of Bioengineering, Rice University, Houston, Texas, USA; 3 Department of Clinical Services, Malawi Ministry of Health, Lilongwe, Malawi; 4 Department of Paediatrics, College of Medicine, Queen Elizabeth Central Hospital, Blantyre, Malawi

**Keywords:** neonatology, global health

## Abstract

**Objective:**

To determine whether a national quality improvement programme implementing continuous positive airway pressure (CPAP) at government hospitals in Malawi improved outcomes for neonates prioritised by an algorithm recommending early CPAP for infants weighing 1.0–1.3 kg (the 50th percentile weight at 30 weeks’ gestation).

**Design:**

The analysis includes neonates admitted with respiratory illness for 5.5 months before CPAP was introduced (baseline period) and for 15 months immediately after CPAP was implemented (implementation period). A follow-up data analysis was completed for neonates treated with CPAP for a further 11 months.

**Setting and patients:**

Neonates with admission weights of 1.0–1.3 kg before (106 neonates treated with nasal oxygen) and after implementation of CPAP (153 neonates treated with nasal oxygen, 103 neonates treated with CPAP) in the newborn wards at Malawi government district hospitals. Follow-up analysis included 87 neonates treated with CPAP.

**Intervention:**

Neonatal CPAP.

**Main outcome measure:**

We assessed survival to discharge at 23 government district hospitals with no significant differences in transfer rates before and after implementation of CPAP.

**Results:**

Survival improved for neonates with admission weights from 1.0 to 1.3 kg treated with CPAP (30.1%) as compared with neonates of the same weight band treated with oxygen during the baseline (17.9%) and implementation (18.3%) periods. There was no significant difference in survival for neonates treated with CPAP during the implementation and follow-up periods (30.1% vs 28.7%).

**Conclusions:**

Survival for neonates weighing 1.0–1.3 kg significantly increased with a nurse-led CPAP service in a low-resource setting and improvements were sustained during follow-up.

## Abstract (precis)

This paper demonstrates the effectiveness of a quality improvement program for the use of early CPAP among neonates weighing 1.0-1.3 kg in Malawi. This nurse-led CPAP program showed a significant improvement in mortality rates that was sustained during the follow-up period.

**What is already known on this topic?**

- Early continuous positive airway pressure (CPAP) is recommended for neonates <30 weeks gestational age but CPAP is often not available in low-resource settings.
- National scale of neonatal CPAP in Malawi significantly improved survival for neonates with respiratory distress.
- When gestational age is unknown, a previously validated algorithm could help identify and prioritise neonates that may benefit from early CPAP.

**What this study adds?**

- This analysis examined whether providing CPAP for neonates prioritised by an algorithm recommending early CPAP for babies weighing 1.0–1.3 kg improved survival to discharge.
- Survival for neonates prioritised for early CPAP significantly increased with a nurse-led CPAP programme in district hospitals in a low-resource setting.
- Improvements in survival were sustained during a follow-up period of analysis.

## Background

Globally, preterm birth is the leading cause of child mortality.^1^ In low-resource settings, most babies with a birth weight below 1.5 kg are premature and mortality is inversely proportional to gestational age.^2^ Babies who are born prematurely have poor thermal control, a weak or absent suck reflex, an inability to contain infections and immature lungs. The lungs are structurally immature and lack surfactant, a lipoprotein that forms a monolayer between air-fluid interfaces, lowers surface tension and prevents atelectasis. If lungs fail to expand properly, there is ventilation–perfusion mismatch and respiratory and metabolic acidosis develop. The work of breathing exhausts the infant who, without help, may die. In a Ghanaian cohort study, mortality was 48 times higher in very low birthweight (VLBW) infants weighing less than 1.5 kg than infants weighing over 2.5 kg.^3^ In a South American group of babies weighing 1–1.5 kg, 74% developed respiratory distress syndrome (RDS).^4^

RDS starts soon after birth and continues to develop over several days. Ideally, once a preterm baby is born, the earlier respiratory assistance is given, the better the outcome.^5^ Respiratory support can be given with continuous positive airway pressure (CPAP), which provides a gentle but continuous flow of mixed air and oxygen to the infant to prevent collapse of alveolar spaces, improve the ventilation/perfusion ratio and reduce the effort of breathing. In high-resource settings, prophylactic CPAP in babies below 32 weeks’ gestation or <1500 g has been associated with reduction in the need for mechanical ventilation and the use of surfactant.^6^ Current guidelines recommend that CPAP should be started at birth for neonates below 30 weeks gestation.^7^ Prophylactic CPAP for VLBW infants in middle income countries in South America reduced mortality rates.^8^

**Figure 1:**
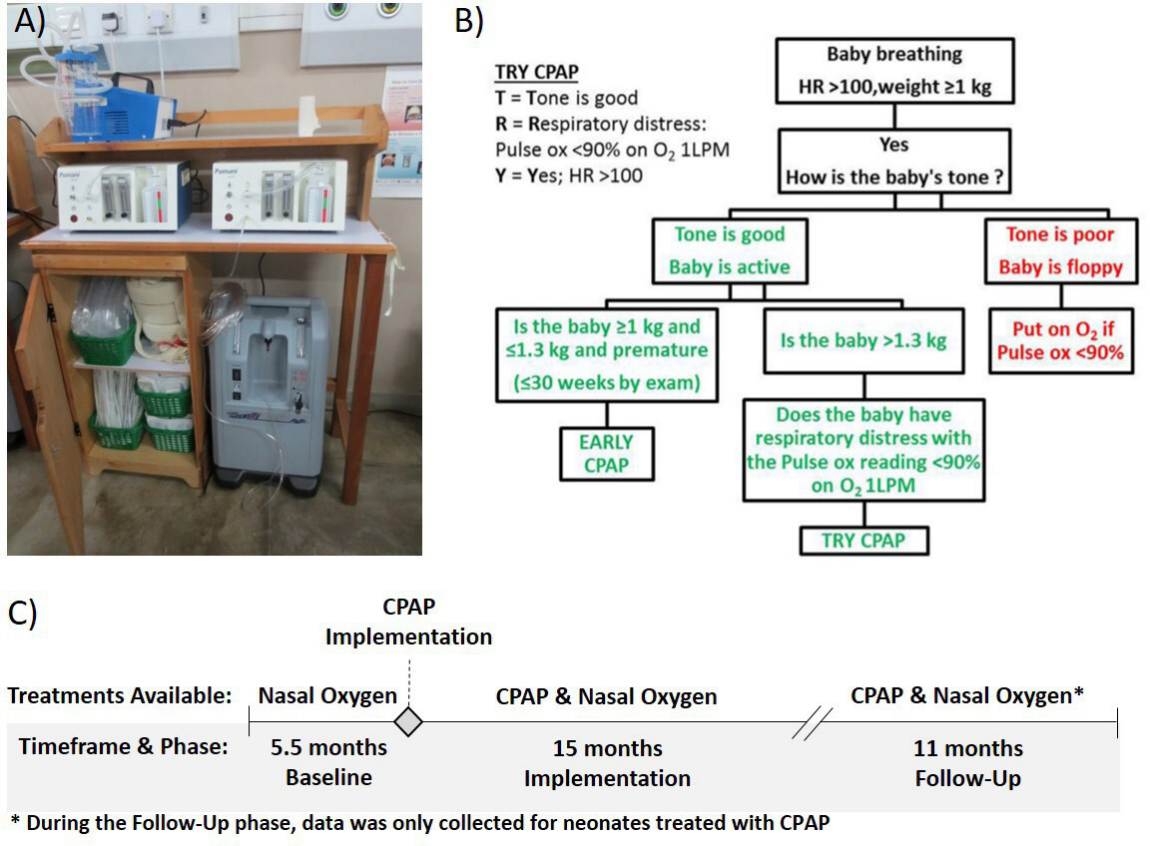
(A) Bundle of equipment and supplies provided with CPAP devices, (B) algorithm for initiation of CPAP in neonates with respiratory illness (reused with permission from Hundalani *et al* 11), (C) study timeline and treatments available during each phase. Figures A and C were created by the authors. CPAP, continuous positive airway pressure; HR, heart rate.

In 2013, we began phased implementation of CPAP to treat neonates with respiratory illness in all government district and central hospitals in Malawi.^9^ CPAP devices along with supporting equipment (oxygen concentrators, suction machines, pulse oximeters, all necessary disposable supplies, a storage cabinet and wall job aids) were installed at each site (figure 1A). Trainings were conducted to emphasise proper patient identification, CPAP device operation, initiation and monitoring of CPAP and weaning patients from CPAP, as well as routine postnatal care of the newborn and the triage of sick neonates.^10^ As part of the initial training, a simple, validated algorithm (figure 1B) using a combination of vital signs, tone and birth weight^11 12^ was introduced to help nurses determine the need for CPAP and ensure efficient and effective use of limited resources in settings where patients’ access to antenatal steroids and chest X-rays was poor, and surfactant and mechanical ventilation were not available. This algorithm has been incorporated into Malawi’s national guidelines for newborn care for diagnosing RDS.^13^ As shown in figure 1B, the first two steps of the algorithm prioritise CPAP treatment for spontaneously breathing infants weighing at least 1 kg with a heart rate (HR) greater than 100 beats per min (bpm) and good tone,^11 12^ as infants with an HR below 100 bpm after initial resuscitation still require active resuscitation,^14^ and neonates with hypotonia are likely to have severe birth asphyxia and are less likely to benefit from CPAP treatment.^15^ As gestational age is often unknown in this setting, the algorithm prioritises early CPAP for spontaneously breathing infants satisfying the first two steps of the algorithm and weighing ≤1.3 kg, which corresponds to the 50th percentile weight at 30 weeks gestation.^16^ This is in accordance with current guidelines that recommend early CPAP for neonates below this gestational age.^7^ The goal of this analysis was to determine whether scale-up of CPAP to district hospitals in Malawi led to improved rates of survival to discharge for neonates with admission weights between 1.0 and 1.3 kg, as prioritised by the algorithm.

## Methods

### Background

As described in detail elsewhere,^9^ a quality improvement programme was implemented from 2013 to 2015 to introduce and monitor CPAP usage and outcomes in the newborn wards at all government district and central hospitals in Malawi where CPAP was not previously available. Baseline training on routine neonatal care and CPAP treatment was conducted for maternal and paediatric staff at each hospital prior to CPAP implementation. Quarterly clinical supervisory visits were conducted by a team consisting of a Ministry of Health (MOH) representative and a physician familiar with CPAP,^9^ and a peer mentorship approach was used to improve and maintain knowledge and skills at each site.^17^

Clinical diagnoses were made in accordance with national guidelines for newborn care in Malawi.^13^ Standard MOH Acute Respiratory Illness (ARI) forms were used for documentation at all government facilities; no additional data collection forms were used. Patient details including dates of birth and admission, admission weight, duration of hospitalisation, discharge diagnosis and outcome were collected from ARI forms for each hospitalised neonate presenting with respiratory illness. An on-site ARI coordinator ensured completion of forms for every qualifying patient. De-identified ARI forms were scanned monthly, and quarterly chart audits were conducted by the MOH ARI team to ensure completion of ARI forms for all patients.

### Data analysis

To evaluate the impact of treatment for neonates prioritised by the algorithm taught in training, neonates with a recorded admission weight between 1.0 and 1.3 kg and treated for respiratory illness at 24 district hospitals where CPAP treatment was not previously available were considered eligible for this analysis. As shown in figure 1C, data were analysed for neonates treated with nasal oxygen during the 5.5 months before CPAP was introduced (baseline period), and for neonates treated with either nasal oxygen or CPAP in the 15 months immediately after CPAP implementation (implementation period). To monitor long-term outcomes on CPAP, a follow-up data analysis was completed for neonates treated with CPAP at all hospitals for a further 11 months between 1 December 2016 and 1 October 2017 (follow-up period).

Transfer criteria varied by facility; no organised transportation system existed between facilities and the decision to transfer was generally determined by proximity to a referral facility. As it was not possible to track outcomes of patients initially admitted to one facility and then transferred to another facility under the approved protocol, one hospital with a significant change in transfer rates between the baseline and implementation periods was excluded from analysis. No neonates from the remaining district hospitals were transferred to another hospital during baseline, and only one neonate was transferred during the implementation period. At the remaining district hospitals, demographics and survival were compared during the baseline and implementation periods for neonates with known outcomes who died or survived to discharge (n=362). Neonates were not included if they left against medical advice (n=17). Similarly, during the follow-up period, demographics and survival for neonates with known outcomes treated with CPAP were analysed (n=87). Neonates were not included if they left against medical advice (n=3) or transferred to another hospital (n=3). Rate of survival was defined as the fraction of eligible neonates with known outcomes who survived to discharge.

Differences in transfer rates and survival between baseline and implementation periods were compared using a two-sided Fisher’s exact test. Differences between continuous variables (ie, weight and age on admission) were assessed using a two-sided t-test for equality of means (unequal variances assumed). Kaplan-Meier survival curves with censoring were calculated during baseline, implementation and follow-up periods; cumulative survival to discharge was compared with a log-rank test. Results were considered significant at the 5% level.

## Results

A summary of the number of neonates weighing 1.0–1.3 kg admitted with respiratory illness at each district hospital during baseline, the number of neonates treated with oxygen or CPAP during implementation and the number of neonates treated with CPAP during follow-up period is available as supplemental information (online supplemental table 1). Transfer rates for neonates 1.0–1.3 kg fell significantly at one district hospital before and after implementation of CPAP (51.9% vs 7.3%, p<0.001); data from this facility were thus excluded from further analysis. No neonates in this weight category were transferred from any of the other district hospitals during baseline; one neonate was transferred during implementation and three neonates were transferred during follow-up period.

10.1136/archdischild-2021-322964.supp1 Supplementary data

Table 1 shows demographic information, outcomes and survival rates for neonates from the remaining district hospitals treated with nasal oxygen during the baseline period (n=106), nasal oxygen or CPAP during the implementation period (n=153 and n=103, respectively) and CPAP during the follow-up period (n=87). Table 2 shows the average admission weight and age on admission for the 449 neonates with known outcomes (ie, died or survived to discharge), stratified by time period and treatment. There were no significant differences in admission weight or age on admission between the four groups.

**Table 1:** Demographic data for eligible neonates with admission weights 1.0–1.3 kg admitted with respiratory distress at district hospitals with no significant changes in transfer rates between baseline and implementation

	Baseline	Implementation (oxygen)	Implementation (CPAP)	Follow-up (CPAP)
Number of study participants	114	161	109	94
Outcome				
Died	76.3%	77.6%	66.1%	66.0%
Discharged	16.7%	17.4%	28.4%	26.6%
Transferred	0.0%	0.0%	0.9%	3.2%
Left AMA	5.3%	3.7%	4.6%	3.2%
Unknown	1.8%	1.2%	0.0%	1.1%
Number of neonates with known outcome (died/discharged)	106	153	103	87
Outcome				
Died	82.1%	81.7%	69.9%	71.3%
Discharged	17.9%	18.3%	30.1%	28.7%
Diagnosis				
Birth asphyxia	16.0%	19.0%	4.9%	3.4%
RDS	45.3%	65.4%	93.2%	87.4%
Pneumonia	0.9%	1.3%	1.0%	1.1%
Meconium aspiration	3.8%	1.3%	0.0%	2.3%
Sepsis	9.4%	5.2%	2.9%	1.1%
No diagnosis	30.2%	17.6%	4.9%	6.9%

AMA, against medical advice; CPAP, continuous positive airway pressure; RDS, respiratory distress syndrome.

**Table 2:** Comparison of weights and age on admission for neonates 1.0–1.3 kg with known outcomes admitted with respiratory distress

	Baseline	Implementation (oxygen)	Implementation (CPAP)	Follow-up (CPAP)
Admission weight (g)	1141±110	1151±108	1144±109	1135±102
Age on admission (days)	0.87±2.71	0.59±2.37	0.75±2.95	0.36±1.45

CPAP, continuous positive airway pressure.

**Figure 2:**
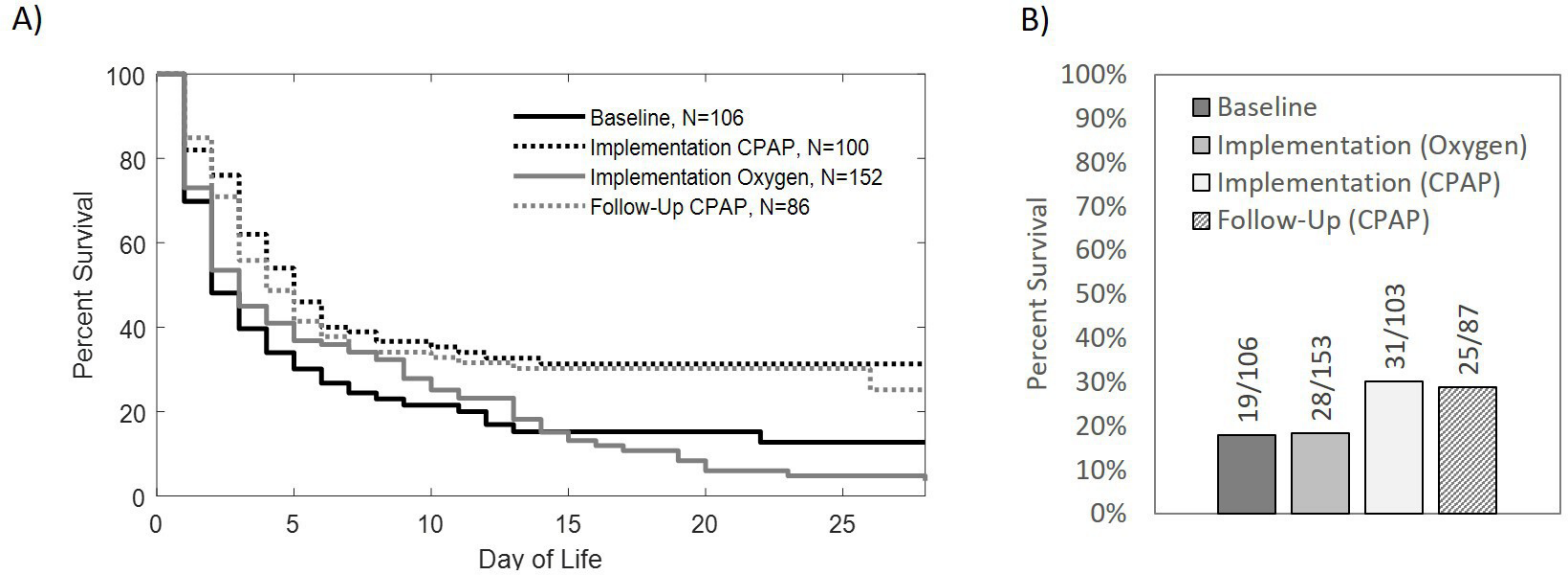
(A) Kaplan-Meier curves with censoring showing 28-day survival to discharge for neonates with admission weights 1.0–1.3 kg treated with oxygen during baseline, oxygen during implementation, and CPAP during implementation and follow-up. Survival rates for neonates treated with CPAP were significantly higher than those treated with oxygen during the baseline (p=0.001) or implementation (p<0.001). Similarly, survival rates for neonates treated with CPAP during follow-up were significantly higher than those treated with oxygen during baseline (p=0.008) and implementation (p=0.002). There was no significant difference in survival for neonates treated with oxygen during baseline or implementation (p=0.82). There was also no significant difference in survival for neonates treated with CPAP during implementation or follow-up (p=0.54). (B) Overall survival to discharge for neonates treated with oxygen during baseline, oxygen during implementation, and CPAP during implementation and follow-up showing similar survival to discharge rates for those treated with oxygen during baseline and implementation (17.9% and 18.3%, respectively). Survival to discharge rates for neonates treated with CPAP during implementation and follow-up were higher (30.1% and 28.7%). This figure was created by the authors. CPAP, continuous positive airway pressure.

Kaplan-Meier estimates of the survival to discharge for neonates treated during the baseline, implementation and follow-up periods are shown in figure 2 stratified by treatment, excluding four neonates from implementation and one neonate from follow-up period that did not have a documented length of hospitalisation. Neonates treated with CPAP during the implementation period had significantly higher survival rates than neonates treated with oxygen alone during the baseline (p=0.001) and during implementation periods (p<0.001). Similarly, neonates treated with CPAP during the follow-up period had significantly higher rates of survival than those treated with oxygen during the baseline (p=0.008) and implementation periods (p=0.002). There were no significant differences in survival for neonates treated with oxygen during baseline and implementation periods (p=0.82) or for neonates treated with CPAP during implementation and follow-up periods (p=0.54).

## Discussion

After CPAP was introduced, the survival of neonates weighing between 1.0 and 1.3 kg receiving CPAP treatment for respiratory distress was significantly higher than those receiving only nasal prong oxygen (p<0.001). Other changes occurred in addition to improved outcomes for neonates receiving CPAP treatment. Overall, diagnosis rates of RDS increased from 45.3% during the baseline to 76.6% during implementation, and the diagnoses of babies given CPAP differed from those who received oxygen. Over 90% of the babies given CPAP were diagnosed with RDS, only 5% were cases of asphyxia and another 5% had no diagnosis, suggesting that after the training and CPAP implementation, diagnoses were more appropriate. This was sustained throughout the follow-up period. On the other hand, two-thirds of babies receiving oxygen after implementation of CPAP had a diagnosis of RDS, 19% had birth asphyxia and 18% had no diagnosis.

Of the neonates that received CPAP treatment, 87.2% commenced treatment within 2 days of admission, with 64% commencing treatment on their day of admission. Given the known advantages of early and prophylactic CPAP, the treatment may have been underused to some extent. This would be similar to a recent report of CPAP to support preterm/VLBW infants in rural Rwanda, where only 59% of CPAP-eligible infants were correctly identified by health providers and only 51.8% were correctly initiated on CPAP.^18^

This study took place in real-world hospital settings and was observational in nature, thus there were inevitable limitations.^9^ As chest X-rays and gestational age as determined by a first trimester ultrasound were rarely available, RDS was diagnosed clinically with the aid of an algorithm. Although some small for gestational age neonates may have been misclassified as premature, a study of Ballard scores done in a central hospital in Malawi suggests that the vast majority of smaller neonates in our setting are premature.^12^ Potential adverse events such as retinopathy of prematurity (ROP), pneumothoraces and nasal trauma were emphasised in training,^10^ but were not tracked as part of this study. However, we did not encounter ROP in the early phases of our programme, and other adverse events were uncommon and limited to nasal bleeds or nasal hyperemia.^19^

Here, survival for neonates receiving CPAP was 1.6 times higher than for those treated with nasal oxygen during implementation, indicating that a nurse-led CPAP service in district-level hospitals had a positive impact on survival for the smallest and most vulnerable infants. Furthermore, this impact was sustained during the follow-up period. However, although overall mortality reduced from 82.1% for those treated with oxygen in the baseline period to 69.9% for those treated with CPAP during implementation, this is still unacceptably high. Though CPAP is now established as an essential tool for care of infants with respiratory distress in all Malawian district hospitals, our results suggest that many infants with RDS who would have benefited might not have received CPAP treatment, and that further impact on survival might be realised by improving patient selection and early initiation of CPAP. Furthermore, these VLBW infants are at risk of multiple complications that cannot be addressed by CPAP alone. Although routine neonatal care was emphasised in trainings, subsequent ward assessments indicated that many of the facilities included in this analysis were lacking functional essential equipment as well as a suitable or separate space for a neonatal unit, and CPAP outcomes improved when these limitations were addressed (Carns J, personal communication 2021. A neonatal ward strengthening program improves survival for neonates treated with CPAP at district hospitals in Malawi). A bundle of simple, reliable and durable equipment to provide holistic care to protect and prevent problems such as hypothermia, hypoglycaemia, jaundice, sepsis and apnoea, to which these infants are also susceptible, is needed to further reduce mortality. However, equipment alone is insufficient; adequate numbers of trained, motivated and experienced nurses are central to the care of VLBW infants, who would benefit from improvements in the capacity for quality monitoring and care. The nurses who diagnose and manage these infants in difficult, under-resourced and understaffed units caring for these smallest and most fragile members of our community deserve our help and admiration.

## Data Availability

Data are available upon reasonable request. Data are available upon request.
