# Supplementary material for: National scale of neonatal CPAP to district hospitals in Malawi improves survival for neonates weighing between 1.0 and 1.3 kg
Source: Arch Dis Child. 2021 Nov 1;107(6):553–7. doi: 10.1136/archdischild-2021-322964 (PMC9125365; doi:10.1136/archdischild-2021-322964)
Supplement: Supplementary data [file archdischild-2021-322964supp001.pdf]

Supplemental Table 1: Eligible neonates with admission weights 1000-1300 g admitted with respiratory distress stratified by hospital

Hospital	Date of Implementation	Number of Neonates Treated with Oxygen (Baseline)	Number of Neonates Treated with Oxygen (Implementation)	Number of Neonates Treated with CPAP (Implementation)	Number of Neonates Treated with CPAP (Follow-Up)
A	11-Jul-13	27	93	17	7
B	15-Aug-13	0	5	2	4
C	3-Jul-13	3	4	3	7
D	4-Jul-13	3	0	2	1
E	21-Aug-13	2	4	4	3
F	15-Mar-14	3	9	4	1
G	5-Mar-14	10	1	4	0
H	1-Apr-14	9	4	1	10
I	24-Mar-14	7	5	3	4
J	14-Mar-14	17	19	8	3
K	1-Apr-14	4	4	2	4
L	12-Mar-14	3	0	1	6
M	5-Mar-14	8	6	3	6
N	18-Mar-14	5	14	2	2
O	6-Jun-15	12	24	0	4
P	31-Aug-15	2	4	14	7
Q	16-Aug-15	5	5	17	4
R	1-Sep-15	4	5	0	4
S	15-Aug-15	1	10	3	6
T	24-Aug-15	8	6	10	5
U	25-Aug-15	2	1	8	4
V	26-Aug-15	1	9	10	4
W	17-Aug-15	3	16	6	5
X	27-Aug-15	2	6	2	0
